# Optimization of pulmonary emphysema quantification on CT scans of COPD patients using hybrid iterative and post processing techniques: correlation with pulmonary function tests

**DOI:** 10.1186/s13244-019-0776-9

**Published:** 2019-10-07

**Authors:** E. de Boer, I. M. Nijholt, S. Jansen, M. A. Edens, S. Walen, J. W. K. van den Berg, M. F. Boomsma

**Affiliations:** 1 Department of Radiology, Isala hospital, Dr. van Heesweg 2, 8025 AB Zwolle, The Netherlands; 2 Department of Innovation and Science, Isala hospital, Zwolle, The Netherlands; 3 Department of Pulmonology, Isala hospital, Zwolle, The Netherlands

**Keywords:** Chronic Obstructive Pulmonary Disease, Emphysema, Respiratory function tests, Computed Tomography, Computer assisted image processing

## Abstract

**Objectives:**

The aim of this study was to assess the effect of hybrid iterative reconstruction and post processing on emphysema quantification in low-dose CT scans of COPD patients using pulmonary function tests (PFT) as a reference.

**Methods:**

CT scans of 23 COPD patients diagnosed with GOLD I or higher were reconstructed with iDose^4^ level 1 to 7 in IntelliSpace Portal (ISP) 6 and 7. ISP7 was used with and without specific denoising filter for COPD. The extent of emphysema was measured as percentage of lung voxels with attenuation < − 950 Hounsfield units (%LAA-950). The correlation between %LAA-950 and PFT, age, BMI, pack years, and the Clinical COPD Questionnaire (CCQ) and Medical Research Council dyspnea scale (MRC) was determined.

**Results:**

Denoising significantly reduced %LAA-950 as was demonstrated by lower %LAA-950 in ISP7 with denoising filter and a significant reduction in %LAA-950 with higher iDose^4^ levels. All PFT except forced vital capacity (FVC) were significantly inversely correlated with %LAA-950. There was a trend toward a stronger correlation at higher iDose^4^ levels. %LAA-950 was also significantly correlated with BMI, GOLD class, and CCQ scores.

**Conclusions:**

Our study showed that hybrid iterative reconstruction and use of post processing denoising can optimize the use of emphysema quantification in CT scans as a complimentary diagnostic tool to stage COPD in addition to PFT.

## Key points

- Denoising reduces the extent of emphysema measured on CT scans.
- Correlation between emphysema and pulmonary function tests increases with higher iDose^4^ levels.
- Emphysema is also significantly correlated with BMI, GOLD class, and CCQ scores.

## Introduction

Chronic obstructive pulmonary disease (COPD) is globally one of the main causes of death. The amount of people suffering from the disease is often underestimated because not all patients with airway symptoms seek medical attention [1]. More attention for early diagnosis of COPD is needed, especially because there is a higher prevalence of pulmonary cancer in COPD patients than in patients without COPD [2]. Moreover, timely treatment may delay disease progression [3].

Currently, COPD is mainly diagnosed based on detection of irreversible airflow limitation in pulmonary function tests (PFT). Airflow limitation is caused by airway narrowing (through inflammation or mucus) and/or emphysema (loss of recoil). Clinically, most if not all COPD patients have combined features of emphysema and chronic bronchitis. Very rarely one encounters a patient with emphysema but little or no airflow limitation [4]. It was shown that the extent of emphysema correlates strongly and significantly with spirometry in quantitative computed tomography (CT) measurements [5]. Therefore, emphysema quantification on CT scans may be an important complimentary tool for the diagnosis and staging of COPD.

To reduce the radiation burden related to CT imaging, advanced iterative reconstruction (IR) techniques during image processing became available that can maintain CT image quality at lower radiation doses compared to filtered back projection (FBP), the standard of reference for reconstruction of CT images. These advanced IR techniques improve subjective as well as objective image quality by reducing noise and artifacts.

**Fig. 1:**
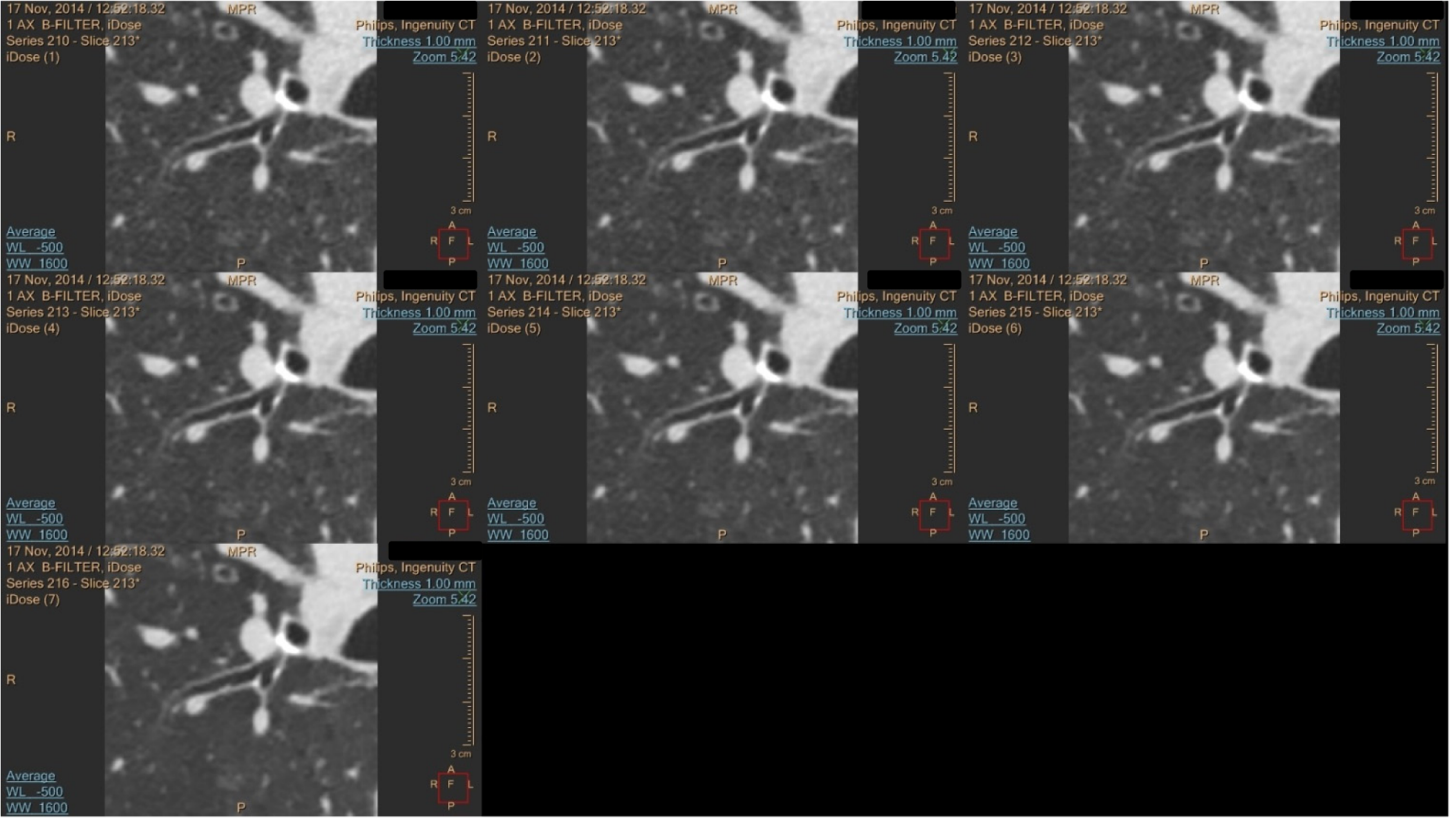
Magnification of the lung parenchyma in a CT scan with the seven different iDose^4^ levels in ISP6: upper row iDose^4^ level 1 to 3; middle row: iDose^4^ level 4 to 6; bottom: iDose^4^ level 7

**Fig. 2:**
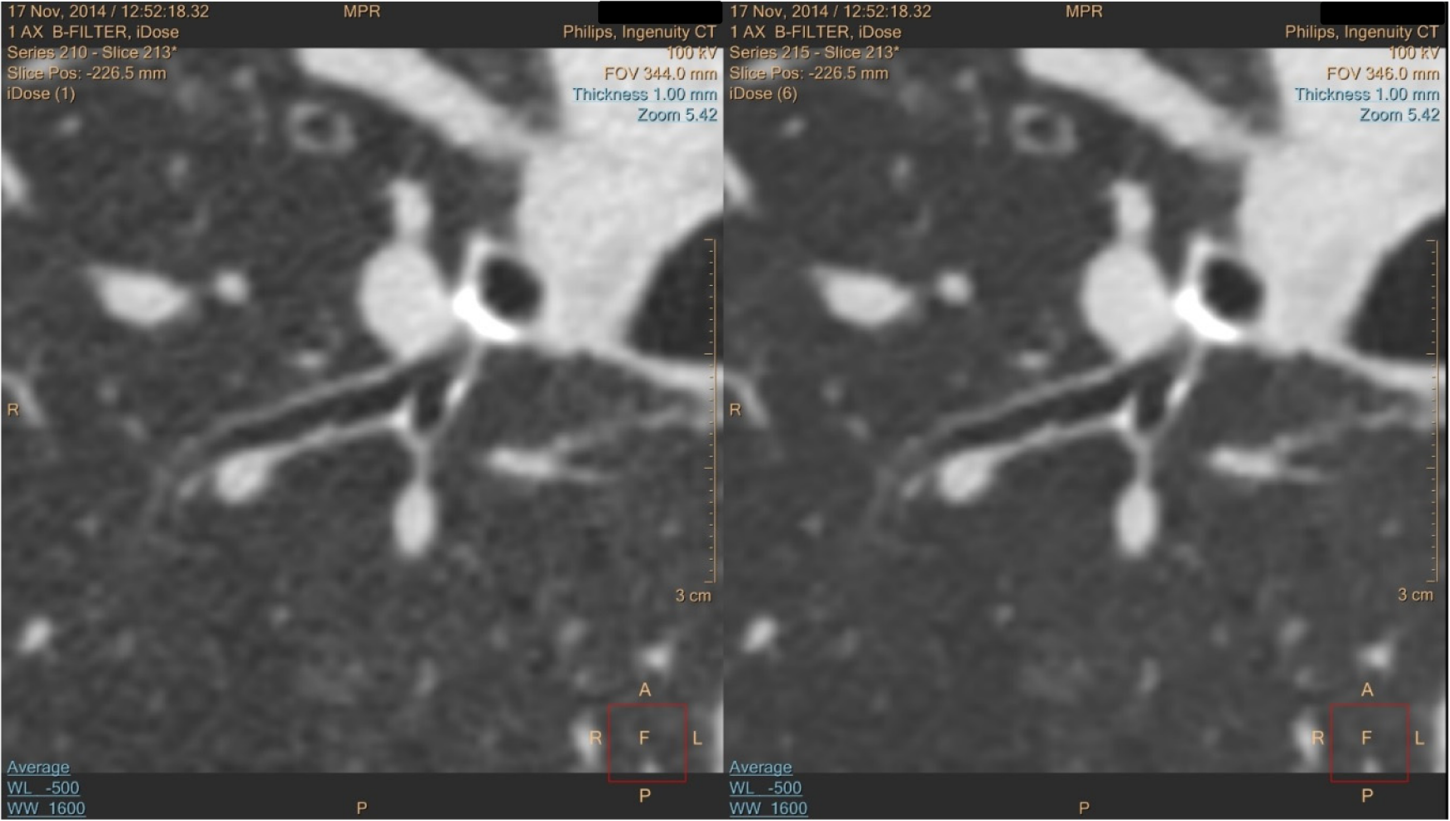
Magnification of Fig. 1, left: iDose^4^ level 1 and right iDose^4^ level 6

In our hospital, we use the hybrid IR algorithm ‘iDose^4^’ (Philips Medical Systems, Best, the Netherlands). This algorithm first identifies and corrects the noisiest raw CT data. These corrected raw data are reconstructed, and subsequently uncorrelated noise in the image domain is iteratively decreased. The level of noise reduction can be adjusted by choosing one of the seven levels (with level 1 corresponding to the least noise reduction, and level 7 to the strongest noise reduction; Figs. 1 and 2) [6–8].

In this study, we aimed to determine which iDose^4^ level resulted in better emphysema quantification in COPD patients using the advanced visualization platform IntelliSpace Portal (ISP) version 6 and 7 (Philips). The correlation with PFT was used as measure of better emphysema quantification.

In ISP7, the COPD advanced visualization application has an optional denoising filter. We also investigated whether this filter could further optimize emphysema quantification in COPD patients.

## Materials and methods

### Study population

We included 23 patients (15 males and 8 females) over 45 years of age that visited the Pulmonary department of our hospital between October 2014 and January 2015. These patients were diagnosed with COPD on the basis of a complete diagnostic path for new COPD patients. This diagnostic path consisted of PFT, questionnaires, and a CT scan. In all patients, the interval between the CT scan and the pulmonary function tests was not more than 6 months. All subjects were asked to fill in the validated Clinical COPD Questionnaire (CCQ). The 10-items of the CCQ comprise three subdomains: symptoms, functional state, and mental state. Items are scored on a Likert scale (range 0–6). The final score is calculated by dividing the sum of all items by 10. Higher scores indicate a worse health status. The Medical Research Council (MRC) dyspnea scale was used to assess the level of disability. It consists of five statements (grades 0–4). The patient is asked which statement describes his/her condition best. Grade 4 represents the most severe category [9]. In addition, the body mass index (BMI), the pack years of smoking, and the number of packages weekly (pack years/52) were assessed. The number of pack years is defined as packs smoked per day × years as a smoker. A pack is considered to contain 20 cigarettes.

### CT data acquisition and image reconstruction

CT examinations were performed using a 64-detector CT (Brilliance iCT, Philips Medical Systems, Best, The Netherlands). All scans were made in supine position with full inspiration breath-hold without spirometric gating. Imaging parameters were kept constant in helical imaging mode: collimation 64 × 0.625 mm (with flying focal spot), 0.5 s rotation time, tube voltage of 100 kVp, tube current of 150 mAs, and pitch 0.798. The effective dose was 1.98 mSv, DLP was 165 mGy/cm, and CTDIvol was 4.2 mGy. Contrast enhancement was not performed and tube current modulation was not used and images were reconstructed at 0.9 mm thickness with 0.45-mm increment using a standard kernel in a 512 × 512-pixel matrix.

Raw CT data of the study objects were reconstructed using iterative reconstruction (IR; iDose^4^ level 1–7; Philips Medical Systems , Best, the Netherlands).

### Quantification of emphysema

Images were analyzed with the COPD advanced visualization application in ISP version 6 and 7 (Philips Medical Systems, Best, The Netherlands). We used ISP7 with and without de-noising filter. The extent of emphysema of the entire lung was in all batches quantified by the percent of lung voxels with attenuation < − 950 Hounsfield units (HU) (%LAA-950). We used specialized software to automatically divide the lungs from the chest wall, mediastinum diaphragm, and airways in the CT images of reconstructed algorithms [10].

### Pulmonary function tests

Pulmonary function tests (PFT) were performed with Geratherm equipment (Geratherm Respiratory GmbH, Germany). During these spirometry tests, the patient was at rest in a seated position. Forced expiratory volume in the first second (FEV_1_) and forced vital capacity (FVC) were acquired according to the European Respiratory Society (ERS) guidelines [11–13] and were expressed as percentage of predicted value. We used the Global Lung Initiative equations 2012 (GLI) as reference values [14]. FEV_1_/FVC ratios were calculated and expressed as percentages.

### Statistical analysis

Statistical analysis was performed using statistical software (IBM SPSS Statistics, Release 22.0: IBM Corp, Armonk NY). Categorical data were presented as *n* (%). Continuous data were checked for normality. Normally distributed data were reported as mean ± standard deviation (SD), data with a skewed distribution as median (25th and 75th percentile). Repeated measures ANOVA with ISP (three levels) and iDose^4^ (seven levels) as within subject factors was used to determine whether %LAA-950 was significantly different between the ISP versions and the seven iDose^4^ levels.

Spearman correlation analysis for nonparametric data was performed for each iDose^4^ level in ISP6 and 7 with and without denoising filter to determine the correlation between %LAA-950 and age, BMI, GOLD, pulmonary function tests, or scores on the CCQ and MRC. *P* values < 0.05 were considered statistically significant.

## Results

The 23 COPD patients (15 males and eight females) included in this study had an average age of 67.8 ± 9.9 years (Table 1). Median body mass index (BMI) was 27.7 kg/m^2^ (21.8–29.6). The majority of the patients was classified as GOLD II (*n* = 10, 43.6%; Table 1). Fifteen of the 23 patients (65.2%) were smokers. The median pack years of all smokers was 30 (20–40) and the median number of packages weekly 0.58 (0.38–0.77). The median pack years of the complete study population was 20.0 (0.0–38.0) and the median number of packages weekly 0.38 (0–0.78). PFT showed impaired lung function as indicated by a mean FEV_1_% predicted of 54.2% (± 22.5), and mean FEV_1_/FVC ratio of 0.47 (± 0.15) (Table 1). The median interval between the CT scan and the pulmonary function tests was 13 days (0–31).

**Table 1:** Characteristics and demographics of the study participants

Demographics	
Sex (male, %)	15 (65.2%)
Age (mean in years, SD)	67.8 (± 9.9)
Anthropometry	
BMI (median in kg/m^2^, p25–p75)	27.7 (21.8–29.6)
Smoking	
Smoker/former smoker (yes, %)	15 (65.2%)
Pack years (median, p25–p75)	20.0 (0.0–38.0)
Packages weekly (median, p25–p75)	0.38 (0–0.78)
Pulmonary function	
GOLD stage (*n*, %)	
1	3 (13.0%)
2	10 (43.5%)
3	6 (26.1%)
4	4 (17.4%)
FEV_1_ (mean in L, SD)	1.57 (± 0.72)
FEV_1_% pred (mean %, SD)	54.2 (± 22.5)
FVC (mean in L, SD)	3.35 (± 1.12)
FEV_1_/FVC (mean, SD)	0.47 (± 0.15)
Questionnaires	
MRC^*n* = 21a^	
0	3 (14.3%)
1	8 (38.1%)
2	1 (4.8%)
3	5 (23.8%)
4	4 (19.0%)
CCQ^*n* = 21a^	2.28 (± 1.09)

*BMI* body mass index, *pack years* packs smoked per day × years as a smoker, *packages weekly* pack years/52, *GOLD* global initiative for chronic obstructive pulmonary disease, *FEV*_1_ forced expiratory volume in 1 s, *FEV*_1_% *pred* forced expiratory volume in the first second as percentage of predicted, *FVC* forced vital capacity, *FEV*_1_/*FVC* ratio of FEV_1_ divided by the forced vital capacity, *MRC* Medical Research Council dyspnea scale, *CCQ* Clinical COPD Questionnaire

^a^Two participants did not fill in the MRC and CCQ

**Fig. 3:**
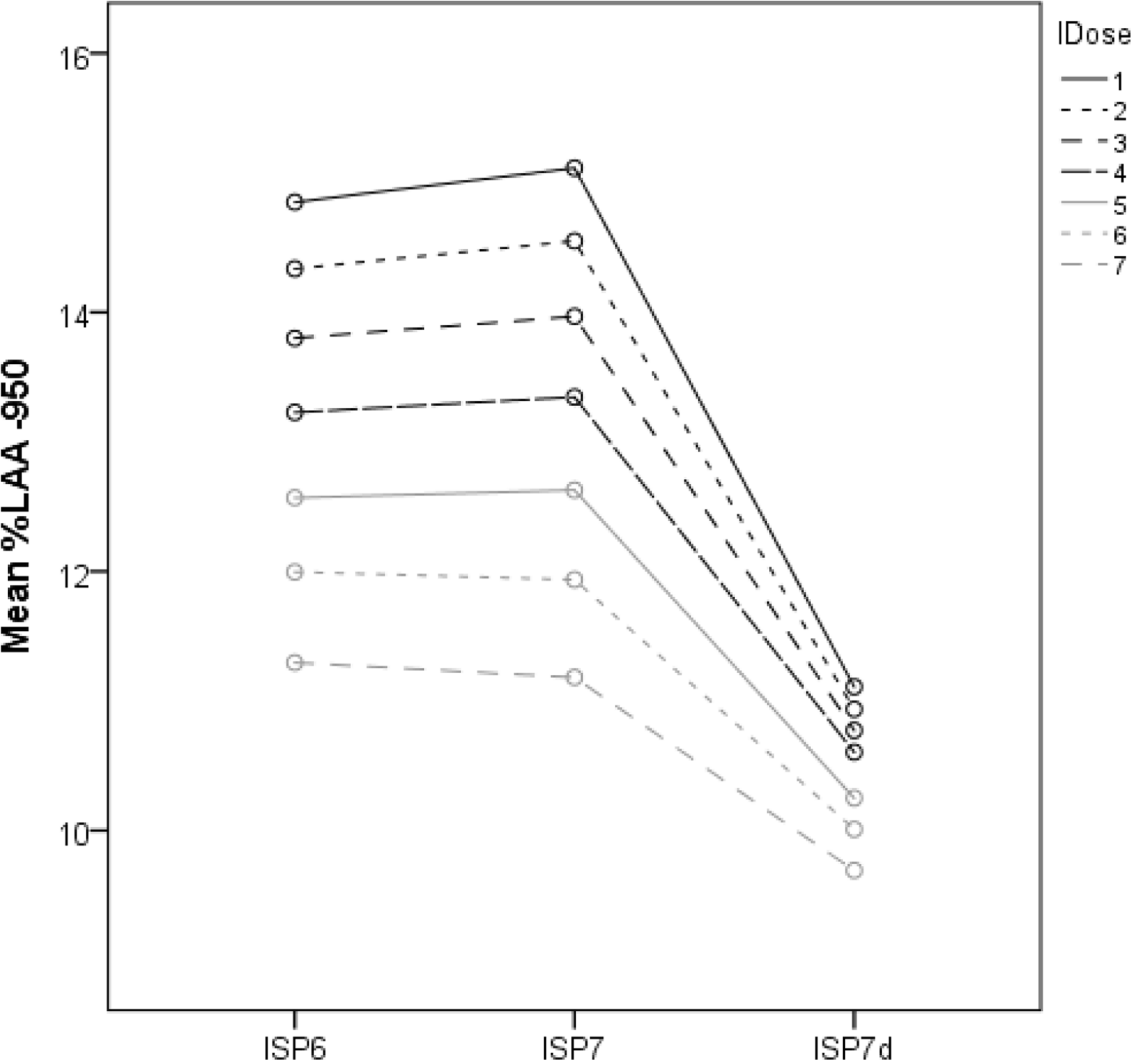
Profile plot of %LAA-950 for the seven levels within ISP6, ISP7 with and without de-noising. %*LAA*-*950* percentage of lung voxels with attenuation < − 950 Hounsfield units, *ISP* IntelliSpace Portal, *ISP7d* ISP7 with denoising

**Fig. 4:**
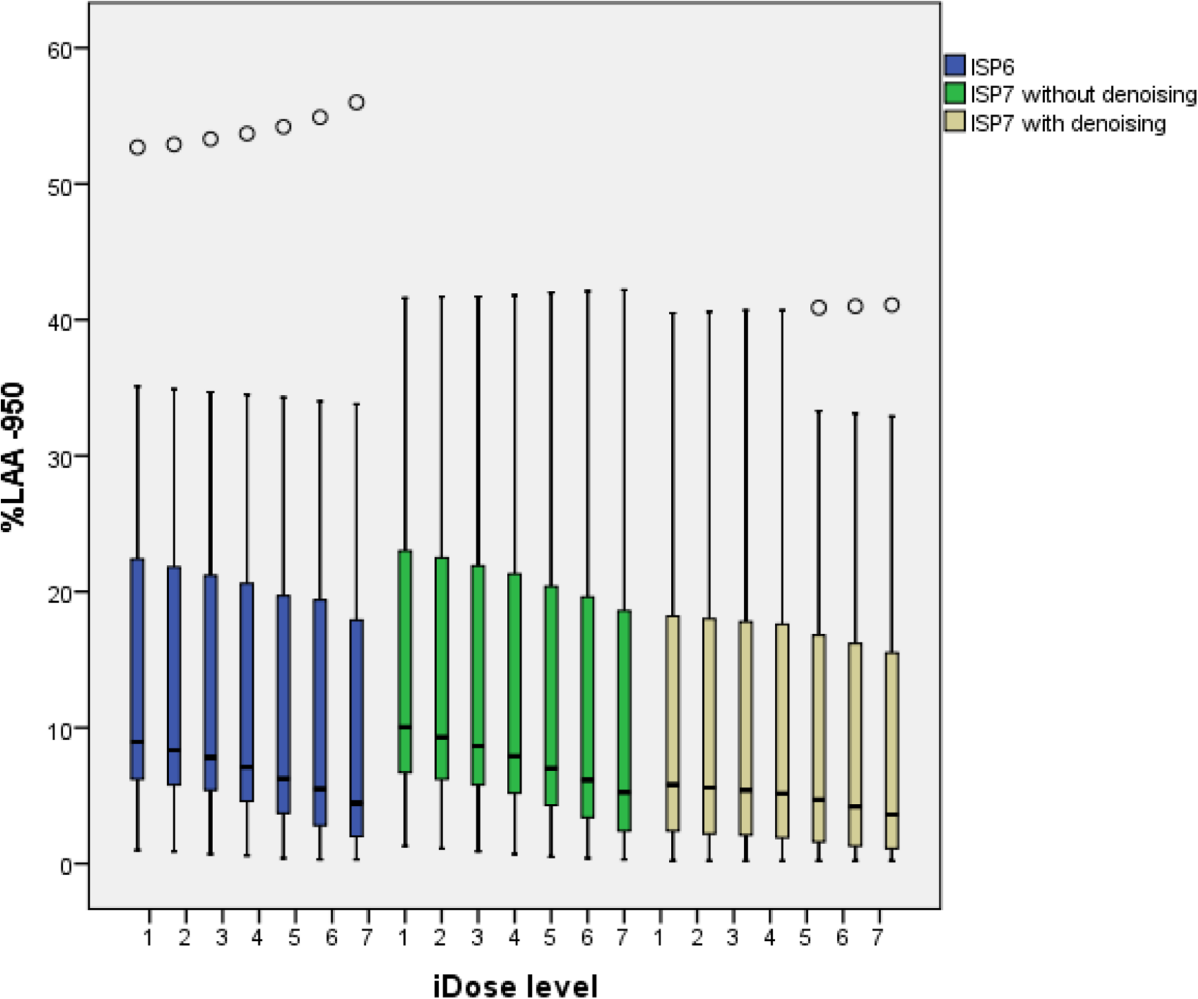
Boxplot of %LAA-950 for the various iDose^4^ levels of ISP6 and ISP7 with and without denoising (*n* = 22, one patient had missing values). %*LAA*-*950* percentage of lung voxels with attenuation < − 950 Hounsfield units, *ISP* IntelliSpace Portal

We compared the %LAA-950 at several iDose^4^ levels of two ISP software versions: ISP6 and ISP7. ISP7 was used with and without denoising filter. Using repeated measures ANOVA, we found a significant difference in %LAA-950 between groups on the level of ISP and iDose^4^ (ISP *p* < 0.001, iDose^4^ *p* < 0.0001) and a strong interaction effect of these two within subjects factors (*p* < 0.0001) (Fig. 3). %LAA-950 significantly decreased with higher iDose^4^ levels for both ISP6 and 7 (with and without denoising) (*p* < 0.001; Figs. 3 and 4). In addition, use of the denoising tool in ISP7 resulted in a significantly lower %LAA-950 for all levels when compared to ISP6 and ISP7 without denoising (Figs. 3 and 4).

We did not observe a significant correlation between %LAA-950 and age, pack years, or MRC scores in all ISP versions. There was a moderate inverse correlation between %LAA-950 and BMI. GOLD stage and CCQ scores were positively correlated with %LAA-950 (Table 2).

**Table 2:** Spearman correlation coefficients

	%LAA-950 per iDose^4^ level						
	1	2	3	4	5	6	7
Body mass index							
BMI ISP6	− .439*	− .439*	− .429*	− .426*	− .418*	− .405	− .416*
BMI ISP7	− .455*	− .450*	− .450*	− .444*	− .433*	− .444*	− .438*
BMI ISP7d	− .424*	− .424*	− .424*	− .424*	− .408	− .423*	− .404
GOLD stage							
GOLD ISP6	.489*	.489*	.489*	.489*	.497*	.513*	.518*
GOLD ISP7	.456*	.467*	.467*	.467*	.492*	.469*	.469*
GOLD ISP7d	.469*	.469*	.469*	.469*	.525*	.492*	.493*
Pulmonary function tests							
FEV_1_ ISP6	− .458*	− .458*	− .464*	− .465*	− .475*	− .507*	− .510*
FEV_1_ ISP7	− .430*	− .442*	− .442*	− .445*	− .471*	− .453*	− .460*
FEV_1_ ISP7d	− .475*	− .475*	− .475*	− .475*	− .509*	− .488*	− .493*
FEV_1_% ISP6	− .551**	− .551**	− .554**	− .556**	− .564**	− .588**	− .591**
FEV_1_% ISP7	− .526**	− .532**	− .532**	− .534**	− .544**	− .539**	− .542**
FEV_1_% ISP7d	− .544**	− .544**	− .544**	− .544**	− .590**	− .574**	− .572**
FVC ISP6	.090	.090	.101	.104	.097	.069	.080
FVC ISP7	.125	.109	.109	.114	.092	.122	.115
FVC ISP7d	.101	.101	.101	.101	.053	.105	.105
FEV_1_/FVC ISP6	− .809**	− .809**	− .820**	− .823**	− .825**	− .838**	− .845**
FEV_1_/FVC ISP7	− .799**	− .800**	− .800**	− .807**	− .812**	− .813**	− .814**
FEV_1_/FVC ISP7d	− .814**	− .814**	− .814**	− .814**	− .834**	− .841**	− .838**
CCQ							
CCQ ISP6	.499*	.499*	.521*	.521*	.531*	.562**	.579**
CCQ ISP7	.507*	.510*	.510*	.510*	.523*	.550**	.560**
CCQ ISP7d	.566**	.566**	.566**	.566**	.550*	.572**	.607**

*ISP* IntelliSpace Portal, *ISP7d* ISP7 with denoising filter, %*LAA*-*950* percentage of lung voxels with attenuation < − 950 HU, *BMI* body mass index, *FEV*_*1*_ forced expiratory volume in 1 s, *FEV*_*1*_% *pred* forced expiratory volume in the first second as percentage of predicted, *FVC* functional vital capacity, *CCQ* Clinical COPD Questionnaire scores

*p < 0.05; **p < 0.001

There was no significant correlation between %LAA-950 and FVC. However, %LAA-950 showed significant inverse correlations with the other three spirometry measures; FEV_1_, FEV_1_% predicted, and FEV_1_/FVC. The strongest correlation was found with FEV_1_/FVC. For all three measures, the negative correlation coefficient slightly increased with higher iDose^4^ levels in all ISP versions. However, there was no difference in correlation coefficients between ISP6, ISP7 with and without denoising filter per level (Table 2).

## Discussion

In this study, we aimed to optimize emphysema quantification in CT scans of COPD patients using hybrid iterative techniques and post processing. PFT outcomes, which are currently the standard for staging COPD, were used as a reference. We compared the results of two ISP software versions; ISP6 and ISP7 (with and without specific denoising filter for COPD).

Previous studies already showed that dose reduction, post processing, and IR can affect the quantitative measurement of emphysema [15, 16]. To the best of our knowledge, this is the first study that showed that the hybrid IR levels of iDose^4^ have a significant influence on the amount of emphysema that is quantified by means of post processing in a low dose scan. The amount of emphysema quantified significantly reduced with higher iDose^4^ levels. The specific denoising filter in the COPD application of the ISP7 module resulted in even lower emphysema quantification at all 7 iDose^4^ levels when compared to ISP6 and ISP7 without denoising filter. Denoising obtained at higher IR levels may have an effect on the final noise statistics of the data, which in turn influences the quantification obtained with a fixed threshold. Other studies also tested post processing of all seven iDose^4^ levels on CT scans of phantoms and patients but only looked at technical variables, e.g., subjective image quality, the contrast-to-noise ratio (CNR), and signal-to-noise ratio (SNR). They did not investigate the effect on tissue quantification [17, 18]. A study on the effects of iDose^4^ on solid pulmonary nodules did not report any differences between iDose^4^ levels 2, 4, and 6 [19].

Our finding that the amount of emphysema quantified depends on the IR used confirms the results of a recent study from Hague and colleagues who observed lower emphysema levels when using a partial IR technique called adaptive statistical IR (ASiR; GE healthcare, Milwaukee, MI, USA) [20]. Unfortunately, iterative techniques from different vendors range from less computationally demanding IR algorithms that reconstruct in the image data domain to IR algorithms that reconstruct in the raw data domain and more advanced hybrid iterative techniques that reconstruct in both domains such as iDose^4^. These dissimilarities in reconstruction result in differences in noise reduction which make it difficult to directly compare the amounts of emphysema quantified between studies [21].

In order to determine which hybrid IR iDose^4^ level would be best for the quantification of emphysema as diagnostic aid for the staging of COPD, the results of the two ISP software versions: ISP6 and ISP7 (with and without denoising filter for COPD) were compared. We correlated the %LAA-950 measured at different iDose^4^ levels in these two software versions with FEV_1_, FEV_1_% predicted, FVC, and FEV_1_/FVC, which are commonly used in diagnosing and staging COPD. We observed a trend toward a stronger inverse correlation of %LAA-950 with FEV_1_, FEV_1_% predicted, and FEV_1_/FVC at higher iDose^4^ levels. The strongest inverse correlation was found between %LAA-950 and FEV_1_/FVC. This is consistent with the findings of D’Anna and colleagues who also reported that percentage of emphysema was inversely correlated with the FEV_1_/FVC ratio in patients with stable COPD of different severity [22]. %LAA-950 was not correlated with FVC.

From our finding that the correlations did not differ between ISP6 and ISP7 with and without denoising filter, we conclude that in clinical practice, both software versions would be equally suitable for diagnosing and staging COPD. However, this does not rule out that other IR techniques and ISP software versions may correlate better with PFT and may thus further improve diagnosing and staging of COPD.

Xie and colleagues performed a large meta-analysis in 2012 to determine the correlation between airflow obstruction and emphysema quantification on CT scans of COPD patients [23]. They also found significant correlations between airflow obstruction parameters in PFT and CT measurements of emphysema. The correlation coefficients that we found in our study were comparable to the pooled correlation coefficients reported in this systematic review [23].

In our study, the extent of emphysema was also inversely correlated with BMI. The finding that COPD patients with the highest emphysema percentages had a slightly lower BMI may be partly explained by the smoking behavior of these patients. Nicotine is known to suppress appetite which can result in lower nutrient intake in smokers. Moreover, smoking increases energy metabolism at rest which may also contribute to lower BMI in COPD patients [24]. As was expected, the extent of emphysema correlated with respiratory symptoms, scored via CCQ.

Our study is limited by the small patient population of 23 patients. However, it proved to be sufficient to detect significant differences and correlations, although the spectrum of emphysema amount was limited. Our data should be confirmed in a larger study population. Moreover, we did not compare the extent of emphysema with diffusion capacity. Previous studies showed a correlation between these variables [22].

The ISP software is continuously improved and new versions are released. Moreover, other emphysema quantification software has become available. These programs and versions start to find their way into clinical trials and routine care. In general, the final image that is used by a certain post processing quantification tool depends on the scanner, radiation dose, and the reconstruction technique. Potential differences in the amount of emphysema measured using various emphysema quantification programs and versions even in CT scans of the same patient may lead to differences in interpretation and ultimately to considerable differences in patient selection and management. The use of a phantom with known reference values would be one of the best ways to standardize emphysema quantification and make it even more suitable as a complimentary diagnostic tool to stage COPD.

## Conclusion

In this within-patients study, we showed that hybrid IR and post processing denoising both affect emphysema quantification in CT scans. The amount of emphysema quantified correlates well with outcomes of PFT indepent of the hybrid IR and post processing denoising applied. Thus, CT scans may be used as additional diagnostic tool to stage COPD.

## Data Availability

The data that support the findings of this study are available from the corresponding author upon reasonable request.

## Abbreviations

%LAA-950: Percentage of lung voxels with attenuation < − 950 Hounsfield unit
BMI: Body mass index
CCQ: Clinical COPD questionnaire
CNR: Contrast-to-noise ratio
COPD: Chronic obstructive pulmonary disease
CT: Computed tomography
CTDIvol: Volume CT dose index
DLP: Dose-length-product
ERS: European Respiratory Society
FBP: Filtered back projection
FEV_1_: Forced expiratory volume in the first second
FEV_1_% pred: Forced expiratory volume in the first second as percentage of predicted
FEV_1_/FVC: Ratio of the forced expiratory volume in the first second divided by the forced vital capacity
FVC: Forced vital capacity
GLI: Global lung initiative equations
GOLD: Global initiative for chronic Obstructive Pulmonary Disease
HU: Hounsfield unit
IR: Iterative reconstruction
ISP: IntelliSpace Portal
MRC: Medical Research Council dyspnea scale
Pack years: Calculated as packs per day times years as a smoker
PFT: Pulmonary function tests
